# Using a Mobile Social Networking App to Promote Physical Activity: A Qualitative Study of Users’ Perspectives

**DOI:** 10.2196/11439

**Published:** 2018-12-21

**Authors:** Huong Ly Tong, Enrico Coiera, Liliana Laranjo

**Affiliations:** 1 Centre for Health Informatics Australian Institute of Health Innovation Sydney Australia

**Keywords:** exercise, fitness trackers, mobile apps, mobile phone, social networking

## Abstract

**Background:**

Despite many health benefits of physical activity, nearly a third of the world’s adult population is insufficiently active. Technological interventions, such as mobile apps, wearable trackers, and Web-based social networks, offer great promise in promoting physical activity, but little is known about users’ acceptability and long-term engagement with these interventions.

**Objective:**

The aim of this study was to understand users’ perspectives regarding a mobile social networking intervention to promote physical activity.

**Methods:**

Participants, mostly university students and staff, were recruited using purposive sampling techniques. Participants were enrolled in a 6-month feasibility study where they were provided with a wearable physical activity tracker (Fitbit Flex 2) and a wireless scale (Fitbit Aria) integrated with a social networking mobile app (named “fit.healthy.me”). We conducted semistructured, in-depth qualitative interviews and focus groups pre- and postintervention, which were recorded and transcribed verbatim. The data were analyzed in Nvivo 11 using thematic analysis techniques.

**Results:**

In this study, 55 participants were enrolled; 51% (28/55) were females, and the mean age was 23.6 (SD 4.6) years. The following 3 types of factors emerged from the data as influencing engagement with the intervention and physical activity: individual (self-monitoring of behavior, goal setting, and feedback on behavior), social (social comparison, similarity and familiarity between users, and participation from other users in the network), and technological. In addition, automation and personalization were observed as enhancing the delivery of both individual and social aspects. Technological limitations were mentioned as potential barriers to long-term usage.

**Conclusions:**

Self-regulatory techniques and social factors are important to consider when designing a physical activity intervention, but a one-size-fits-all approach is unlikely to satisfy different users’ preferences. Future research should adopt innovative research designs to test interventions that can adapt and respond to users’ needs and preferences throughout time.

## Introduction

Physical inactivity has been identified by the World Health Organization as a global public health problem, emerging as the fourth leading risk factor for global mortality [1]. Research has shown that physical inactivity increases the risk of many chronic diseases—most notably, type 2 diabetes, coronary heart disease, and colon cancer [2]. Nearly a third of adults worldwide are insufficiently active [3], highlighting the need for effective health interventions to change behavior and promote physical activity.

It is widely acknowledged that behavior change is a challenging process. The success of behavior change depends not only on an individual but also on social and environmental factors [4,5]. Behavior change interventions are usually complex (ie, involving several interacting components), which makes it hard to identify what is effective in changing a particular behavior, for whom, and in what context [6-8]. Several taxonomies for behavior change techniques (ie, the active components in health behavior change interventions) have been developed [9,10] in an attempt to isolate and identify the most effective components of interventions. For physical activity promotion, some behavior change techniques seem to be particularly relevant such as self-monitoring of behavior, goal setting, and social support [11,12]. In addition, the mode of delivery of the intervention is equally important, as it can influence its acceptance, dissemination, and long-term use [8,13].

The use of technology in the delivery of behavior change interventions has potential in promoting their success and diffusion. Notably, mobile health (mHealth) interventions, involving mobile apps and wearable devices, can reach individuals continuously, enabling the self-monitoring of health and physical activity data [14] and the tailoring of intervention components in real time [15]. In addition, Web-based social networks seem to hold great promise, as they can help address social processes related to behavior change such as social support and social comparison [16,17]. Given their potential, interventions combining mHealth technologies and Web-based social networks might be particularly effective in promoting physical activity.

To date, a few qualitative studies have sought users’ attitudes and views on the use of mHealth technologies and Web-based social networks for physical activity promotion [18-22], with most focusing on just one of these technologies. This limits the ability of researchers and developers to assess whether these 2 technologies can work in synergy. In addition, it remains unclear which behavior change components are most effective and which are considered more engaging by consumers [23]. The aim of this study was to explore individuals’ perspectives before and after using a mobile social networking app for physical activity promotion. Specifically, we were interested in exploring potential barriers and facilitators to engagement with the intervention, as well as the behavior change techniques and delivery features considered important by users to promote physical activity. This research will help guide the future development of interventions and public health initiatives that could be more effective in influencing physical activity.

## Methods

### Study Overview

This study is part of a larger mixed-methods feasibility study on the use of a social networking mobile app to promote physical activity and weight management [24]. Given the importance of physical activity and its impact on weight management [1-3], this paper focused specifically on factors influencing physical activity. This study adheres to the COnsolidated criteria for REporting Qualitative research checklist for reporting qualitative research (Multimedia Appendix 1) [25]. This study protocol was approved by the Macquarie University’s Human Research Ethics Committee for Medical Sciences (reference number: 5201600716). The authors declare that the data supporting the findings of this study are available within the paper and its supplementary information files.

### Study Setting and Participants

This study was conducted at Macquarie University (Sydney, Australia). We recruited 55 participants, mostly university staff and students, using purposive sampling techniques through several channels, including posters around campus, website information, social media, and an email newsletter. Eligible participants were healthy adults with sufficient English to understand and participate in the study; aged between 19 and 35 years; who planned to be living in Sydney for the duration of the study; and owned a mobile phone (iOS or Android) with internet access. The exclusion criteria included pregnancy; body mass index (BMI) <17; prior history of eating disorders; or having diabetes or other comorbid conditions that could impact the study participation (eg, severe mental illness and end-stage disease).

For a 6-month period, participants were asked to use an intervention bundle (detailed below). Interviews were conducted pre- and postintervention, with the aim of assessing participants’ perspectives on the use of social networking and mHealth interventions to promote physical activity. Of 55 initial participants, 45 returned for the final interviews.

### Intervention Description

The intervention bundle was composed of a mobile social networking app (named “fit.healthy.me”), a fitness tracker (Fitbit Flex 2), and short message service text messages and emails [24]. The mobile app “fit.healthy.me” consisted of several features—“My measures,” “My team,” “Social forum,” and “Private message”—which directly supported different behavior changes techniques (self-monitoring, social support, and social comparison). Specifically, “My measures” provided a summary of the number of steps, weight, and BMI. “My team” was a platform for participants to visualize and compare their steps with others. “Social forum” and “Private message” were designed for individuals to network with other users and provide and receive social support.

To enable the automation of self-monitoring, the app was integrated with the Fitbit Flex 2 fitness tracker, through the Fitbit Application Programming Interface. Reminders to wear the trackers and check the app were sent to participants every 2 weeks in the form of short message service text messages and emails. Table 1 provides a detailed description of the modes of delivery and features of the intervention, and Multimedia Appendix 2 shows the screenshots of the “fit.healthy.me” app.

**Table 1:** Intervention description.

Modes of delivery	Features	Behavior change techniques^a^
fit.healthy.me app	My measures	Self-monitoring of behavior (ie, physical activity)
	My team	Social comparison
	Social forum	Social support Social comparison
	Private message	Social support Social comparison
	My journey	Instruction on how to perform the behavior
Fitbit Flex 2	Fitness tracker	Self-monitoring of behavior (ie, physical activity)
Texts and emails	Reminders	Prompts or cues

^a^Classified according to the behavior change technique taxonomy developed by Michie et al [26].

### Interview Procedure

Prior to study commencement, an interview guide (Multimedia Appendix 3) was developed and pilot-tested. Participants were invited to attend the initial study session at the research center, where they received information about the purpose of the study, signed the consent form, and filled in a questionnaire about their demographic characteristics and smartphone usage (eg, the type of smartphone used and hours per day spent using the smartphone).

In the preintervention session, 55 participants attended a brief individual interview (10-15 minutes) in which they were asked about perceived facilitators and barriers to physical activity and their views on the potential advantages and disadvantages of the mobile app and wireless devices (fitness tracker and scale). The content of the preintervention interviews was summarized and used as prompts for discussion in the postintervention sessions.

In the postintervention session, we conducted 32 individual interviews and 5 focus groups with 13 participants (20-45 minutes); data saturation was reached. While the interviews allowed us to understand individual perspectives, the focus groups enabled us to explore group differences and similarities [27,28].

At the postintervention sessions, participants talked about their experiences regarding the use of the intervention and provided suggestions on the devices and the intervention. Furthermore, semistructured interviews were conducted by 2 researchers with expertise in qualitative methods. Field notes were taken throughout the interviews.

### Data Management and Analysis

With participants’ consent, the interviews were recorded and transcribed verbatim, and transcripts were analyzed in Nvivo 11 (QRS International Pty Ltd., Melbourne, Australia). The data were analyzed using thematic analysis techniques [29]. Specifically, the transcripts were explored using the inductive analysis to identify themes and patterns [29]. First, we open-coded the transcripts to identify all important aspects related to the research questions. Subsequently, by scrutinizing and comparing different data and codes (ie, constant comparison), we pinpointed concepts that seemed to cluster together [30]. Informed by engagement with the literature, we identified the similarities, differences, and general patterns in the open codes, to fill in underdeveloped categories, narrow excess ones, and organize them into major themes [30,31].

## Results

### Sample Characteristics

Table 2 summarizes participants’ demographic characteristics. At baseline, 51% (28/55) participants were females; the mean age was 23.6 years. On average, participants spent 5.6 hours daily using smartphones, and 89% (49/55) participants stated that they frequently used social media. Of all, 76% (42/55) participants were university students.

### Summary of Results

We found the following 3 types of factors emerging from the data as influencing user engagement with the intervention and physical activity levels: individual, social, and technological. At the individual level, participants mentioned that goal setting, self-monitoring, and feedback were important for their physical activity. At the social level, social comparison and the connection with other users in terms of familiarity and similarity were considered motivating. Finally, at the technological level, automation and personalization were considered to be facilitators, while technological limitations were observed as reducing user engagement. The following sections discuss each of these themes in detail, with illustrative quotations (Textboxes 1-3).

**Table 2:** Baseline sample characteristics (N=55).

Characteristics		Value
Age, mean (SD)		23.6 (4.6)
Female gender, n (%)		28 (51)
Weight, mean (SD)		78.1 (22.3)
BMI^a^ (kg/m^2^), mean (SD)		26.5 (6.8)
**BMI categories^b^, n (%)**		
	17-18.49	3 (6)
	18.5-24.9	24 (44)
	25-29.9	15 (27)
	≥30	13 (24)
Steps/day, mean (SD)		9937 (3527)
**Marital status, n (%)**		
	Single	27 (49)
	In a relationship	22 (40)
	Married or de facto	6 (11)
Daily smartphone use (hours), mean (SD)		5.6 (3.4)
**Most used apps^c^, n (%)**		
	Social media	49 (89)
	Fitness apps	6 (10)
**Occupation, n (%)**		
	Student	42 (76)
	Other	13 (24)
**Smartphone, n (%)**		
	iPhone	36 (66)
	Samsung	6 (11)
	Other	13 (24)

^a^BMI: body mass index.

^b^According to the World Health Organization, a BMI of <18.5 is classified as underweight, 18.5-24.9 as normal, 25-29.9 as preobese, and ≥30 as obese [32].

^c^Most used apps—options are not mutually exclusive.

### Individual-Level Factors Influencing Physical Activity

#### Self-Monitoring

Self-monitoring was deemed important by many users, as it increased their awareness of activity levels and performance, as well as enabled them to review their progress over time and better plan their exercise (Textbox 1, quotes 1 and 2). Some users indicated that even though self-monitoring was important, knowing the daily number of steps was not sufficient, as they were doing other types of exercise. Thus, they would prefer to measure parameters that were relevant to the type of activity they did (Textbox 1, quotes 3 and 4).

Other than physical activity, users also expressed the desire to monitor a wide range of health-related information (eg, sleep). By having multiple types of information about themselves, users felt they could get an overall view of their daily patterns, and how external factors (eg, family, jobs, and study) affected their health and well-being (Textbox 1, quote 5).

#### Goal Setting

Many participants expressed that they benefited from goal setting. They believed that setting a goal (eg, 10,000 steps daily) kept them accountable for their physical activity performance and motivated them to reach that goal. Participants indicated that goal setting and self-monitoring complemented each other because, without self-monitoring, they would have no way of knowing whether their goals had been achieved (Textbox 1, quote 6). In addition, many participants expressed the desire to be able to personalize their goals to fit with their ability and daily routines, rather than having a standard goal (Textbox 1, quote 7).

#### Feedback on Behavior

For many users, the feedback on progress toward goals was particularly encouraging; knowing that they were close to reaching their goals would motivate users to do more physical activity, while being notified of goal achievement gave them positive emotions (Textbox 1, quotes 8 and 9). Nevertheless, some participants mentioned that knowing they had not achieved their goals also brought on some negative feelings such as disappointment or guilt (Textbox 1, quote 10).

### Social-Level Factors Influencing Physical Activity

#### Social Comparison

Participants mentioned that comparing themselves with other users encouraged them to be more engaged with the intervention, as well as to be more physically active (Textbox 2, quotes 1 and 2). One interesting aspect was that comparisons with higher, lower, or similar standards of physical activity (upward, downward, and lateral comparisons in accordance to [33]) had different effects on performance, according to participants. Most users said that they preferred to compare themselves against higher performers because that motivated them to try to learn their strategies and be more physically active, to beat the top level (Textbox 2, quote 3). Other users mentioned that they would like to compare themselves to both similar and higher standards (Textbox 2, quotes 4 and 5). On the other hand, some participants mentioned that comparison to higher standards could be rather demotivating and confronting, especially when they failed to achieve as many steps as others. Instead, those users preferred comparing themselves with lower standards, which gave them a sense of confidence and assurance that they were on the right track (Textbox 2, quotes 6 and 7).

#### Familiarity With Other Users

For many participants, social comparison and providing social support did not hold much meaning if they did not personally know other users. Many suggested that they were more likely to be engaged if they were “familiar” with other users (eg, if other users were their real-life social connections; Textbox 2, quotes 8 and 9). On the other hand, some participants mentioned that they did not necessarily need to know other users in real life; however, they needed to have some information about other users such as their lifestyle, fitness goals, or the types of activity they did, which could form the basis for social comparison (Textbox 2, quotes 10 and 11).

#### Similarity With Other Users (Homophily)

Other users did not stress the importance of “familiarity”; instead, they described a preference to share data within a social network of people who shared *similar* attributes or goals to them (a phenomenon known as “homophily” [34]). Particularly, some participants preferred to connect with users who had similar BMI or were doing the same type of physical activities (Textbox 2, quotes 12 and 13). In addition, a lot of participants emphasized the importance of having a similar goal, as it might facilitate more meaningful comparison and discussion on PA strategies (Textbox 2, quotes 14 and 15).

**Illustrative quotations for individual-level factors that influence participant engagement and physical activity.**

Self-monitoring of behavior

- Quote 1: The important part for me is [keeping track] – I know I'm going beyond the average, like the normal number of steps for a person […] - it makes me more motivated. (Female, 24)
- Quote 2: I could use the data, so I know how [many] steps for one run, or how long I take for one run. It helps me to evaluate how [many] runs I could actually do, or what should be my targets for next day. (Male, 24)
- Quote 3: I climb now […] I'm actually looking for a watch or something that can measure altitude, it will be more interesting because I'd get to see how far I've climbed. (Female, 20)
- Quote 4: [I do] martial arts, so it’s not so much running and movement. I want to have heartrate, it’d probably be a bit more useful. (Male, 20)
- Quote 5: I realized because of work pressure, in fact, I’m doing two jobs right now […] my average sleep has gone down. (Male, 27)

Goal setting

- Quote 6: There was a goal to reach every day. It kept me motivated […]. I would feel bad if I'm not wearing the [Fitbit]. It was like an additional limb in my body sort of thing.” (Male, 27)
- Quote 7: I want to set my own goals each day […]. Some days I'm more active than other days. On those days, I'll automatically reach 10,000 steps in […] one session alone. But if I changed [the goal] to 20,000 steps then […] it would not really [be] achievable on the days that I don't do that much physical activity. If you could tailor the steps per day, then the motivation would be continuous. Because the motivation only works if I get close up to the end. (Female, 20)

Feedback on behavior

- Quote 8: Because I work long hours, I would reach 10,000 steps at like 10am. It always made me feel good when it vibrated and all the colors everywhere. I was like, yes! (Female, 20)
- Quote 9: When I […] got 80% of my goal, [I would just] go aimlessly for a walk. So that was getting me to walk more. Solely because I was on 80% and I wanted that 100%. (Female, 20)
- Quote 10: It sorts of guilt-tripped a bit. When I’d see it and I was like oh, I’d only done so many steps today. (Female, 19)

#### Participation From Other Users

Participation from other users was important for people to engage with the social network component of the intervention. Many users described attrition as a “domino effect”—once a certain number of people stopped using the app or the wearable tracker, other users subsequently felt less motivated to use the technology (Textbox 2, quotes 16 and 17).

**Illustrative quotations for social-level factors that influence participant engagement and physical activity.**

Social comparison

- Quote 1: It gives me positive reinforcement at the same time because…I’m at the top chart of the steps. It kind of motivates me to stay on that level of rank and in general it motivates me because I can see if I’m doing well or not. I compare myself with the others. (Male, 24)
- Quote 2: I find competition helps me to regularly exercise often by going for runs with friends or family or competing in team sports.…Other people can see [your effort] and keep you accountable to your fitness goals. There’s also that element of showing off…and also being able to see how other people exercise and then try to match them. (Male, 23)
- Quote 3: I probably look up more.…A lot of my days, I get up to 17,000 steps. So, I don’t look down, I’d look up and be like, “Oh, why are those people getting 21,000 steps? I need to get 21,000 steps.” (Female, 24)
- Quote 4: I would obviously want my comparison to be done with somebody who is exactly like me, or similar in certain ways. It gives me some kind of happiness that I’m achieving my goals in comparison to this person. It’s like a competition. It’s like scoring 87 and the other person is scoring 84.…Then I would also want to know the person who has got a 96 and why did he get a 96?…If you want to achieve 100, you want to know where you went wrong and what did you do right. But I don’t want to compare with a person who got a 40. (Male, 27)
- Quote 5: I was probably competing to the person closest in terms of kilometers that we were doing. It was interesting to see what they were doing and how they progress…. I tried to beat them every day. (Male, 21)
- Quote 6: Being compared to other people was a bit shocking—I was [at] the end of the group, so it was a bit demotivating. (Female, 20)
- Quote 7: If I’m having more steps than others, I feel motivated, and know that at least I keep myself healthy. (Female, 24)

Familiarity with other users

- Quote 8: It’s like, I don’t really know anyone [in the study] and then…the fad of comparing yourself against people wears off; I did try and use it a little bit more, but it was just like because you don’t know anyone, you forget about it.…If it was in a group of my friends, we probably would’ve been checking it weekly. (Female, 24)
- Quote 9: I guess not knowing what they do…—whether they worked or whether they were students— not knowing that, it’s a bit hard to…compare because there’s all these variables. Also, because I really didn’t know them, I didn’t feel obliged to try to motivate them at all in any way. I guess with friends—and if I got to know them at all— yeah, I might have done that. (Male, 30)
- Quote 10: [I’d like to see] more information about the kind of fitness people are doing. For example, someone has done 20,000 steps in a day, which is a huge amount, then give me a basic idea of what that person has done to get to that goal. (Female, 19)
- Quote 11: If everybody [had a] profile, maybe it [would be easier] to make friends. At the beginning I thought “Maybe I can [make a] friend and we can train together to lose some weight.” (Female, 34)

Similarity with other users

- Quote 12: I think it would help if you had people…with a similar body type doing similar things that would suit you more. (Female, 23)
- Quote 13: I like that you could go through and track people who were similar to you…. I went and found people with similar BMI. I’m happy to track myself against similar people and see how many steps [they’ve done]. (Female, 24)
- Quote 14: Everyone’s goal might be different. So, you need to group people with similar goals together. …I would want to compare myself to somebody who [has similar goals] and is using it on a daily basis like me.” (Male, 27)
- Quote 15: Having a goal section where people say whether they want to gain or lose weight would be good. Then all people who want to lose weight can get together and talk about it. (Male, 20)

Participation from other users

- Quote 16: It was a bit like a domino effect, so after about two months you could see that 20 to 30 per cent had zero [steps]. It felt like people weren’t using the app, so there was no reason for me to use it as well. (Male, 22)
- Quote 17: There’s no number of steps [from some people] sometimes. It can be a little demotivating when you see a lot of zeros…It’s like are they taking this seriously? (Male, 24)

### Technology-Level Factors Influencing Physical Activity

#### Technological Facilitators of Engagement and Behavior Change

##### Automation

Many participants found that using the wireless tracker and scale in combination with a mobile app offered many advantages. Specifically, wireless devices provided an automatic way for users to collect and self-monitor personal measurements, and their integration with the mobile app provided a user interface platform for participants to visualize those data and to review progress (Textbox 3, quotes 1 and 2).

##### Personalization

Many users mentioned that having personalized information and services would also support long-term engagement, as they could offer the advantage of providing relevant information tailored to each specific user, thus eliminating the cognitive burden of dealing with information overload. Many users described that personalization should go beyond the content generated by the system and extend to the provision of relevant services (eg, suggestion of exercise routines; Textbox 3, quotes 3-5).

#### Technological Barriers to Continued Usage

##### Additional Workload

As time went on, many users described the feeling that the novelty of the technology had worn off, and they started to think of it as a chore. Even apparently simple tasks like charging the devices were seen by participants as an extra burden in their already busy daily routines (Textbox 3, quotes 6 and 7).

##### Technical Problems and User Experience

Technical problems were often described as a common cause for attrition (Textbox 3, quote 8). In addition, user experience factors, such as the design aspects of the interface and its usability, were reported as important aspects of engagement and continued use (Textbox 3, quotes 9 and 10).

**Illustrative quotations for technological-level factors that influence participant engagement and physical activity.**

Technological facilitators of engagement and behavior change

- Quote 1: I enjoyed how [the wearable tracker] linked with the app, and then on the app you could track how many steps you [did]. […] With the scale as well, the scale was able to track my weight and then it gives you a trend line to show how you're doing, so I enjoyed that as well. Having the combination of the tracker, the scale and the app was really good. (Male, 22)
- Quote 2: I like the [Fitbit] app. It integrates so well, so you wear your [tracker] and then [the app] tells you [how many] exercises you’ve done in a week, your steps, sleep. (Female, 31)
- Quote 3: [Having health information] would be good, but it has to be personalized or customized to me, (…) my body type, […] not like a general advice like [what is] BMI etc. […] A lot of people can read about general information; but if it's personalized to you or customized to your needs, it's going to be more interesting and more reliable […]. (Male, 24)
- Quote 4: I liked that at the end [of a fitness video], you can put a smiley face on how difficult it was. Based on my reaction, I want the app to give me recommendations on what types of exercises I should do. So, it was tailored to me, according on my reaction. (Female, 20)
- Quote 5:
  - Male: Whether to have one or multiple buddies, the choice depends on what works for the person. Maybe you can personalize it in some way. Maybe you can elect I want only one partner, or I want to be put in a group. (Male, 20)
  - Female: It is like gym training session, you can have private sessions, you can have small group sessions, or you can have a class session and you choose which one is best for you. The same with the app and your buddy. (Female, 20)

Technological barriers to continued usage

- Quote 6: The charge lasted three days, and because I had such a busy schedule, charging it again [was] such a big chore. So, it would then just sit for another week and I’d get a [reminder] email and then I would plug it in […]. I was doing so many things, so remembering to charge it became a challenge. (Male, 33)
- Quote 7: After a first couple of months, it started to feel more like a chore to do. I got into the thinking “I had to [check the app] everyday” as opposed to “I want to do this every day to keep track of my weight”. Then university started, and things started getting busy. (Male, 22)
- Quote 8: The battery was discharging very quickly. In the morning it was telling me that I had achieved my goals when I just started the day. (Female, 20)
- Quote 9: I liked the social comparison feature in fit.healthy.me, but it’s hidden in several menus. I liked the Fitbit app better—the design is certainly more elegant. (Female, 26)
- Quote 10: I checked the Fitbit app more than the fit.healthy.me app. I think the reason was because the Fitbit app was much sleeker, looks nicer and more inviting and easier to use. (Female, 20)

## Discussion

### Principal Findings

This study explored users’ perspectives regarding facilitators and barriers in using mobile social networking interventions to promote physical activity. The following 3 categories of influencing factors emerged: individual, social, and technological. At the individual level, behavior change techniques, such as goal setting, self-monitoring, and feedback, were suggested as important for user engagement in physical activity. At the social level, social comparison, familiarity, and similarity with other users were mentioned as motivating aspects. Finally, automation and personalization were highlighted as technological facilitators, enhancing the delivery of both individual and social aspects of the intervention. However, some technological limitations were also found to be barriers to user engagement.

### Comparison With Previous Literature

Our findings suggest that the success of a behavior change depends on a range of factors, including both individual and social aspects. These findings are in line with other behavior change theories, namely the social cognitive theory [4], and the Capability Opportunity Motivation—Behavior model [5]. Both theories suggest that even though several behavioral factors (eg, self-regulation [35], capability, and motivation [5]) are largely dependent on individuals, external factors (eg, peer modeling [4] and environmental structure [5]) can arise from the physical or social environments to prompt behavior. Hence, it seems sensible to integrate both individual and social aspects of behavior change in physical activity interventions to increase their long-term success.

In line with our results, behavioral informatics interventions (eg, a mobile social networking app, connected with a fitness tracker) can facilitate the delivery of both individual and social aspects in physical activity interventions [8]. Specifically, fitness trackers can automate the self-monitoring of behavior and connect to mobile apps with social features, allowing users to not only view their progress but also continuously benefit from social support [23,36]. To date, one qualitative study has examined how wearable trackers, mobile apps, and Web-based social networks may interact, finding that social support from Web-based networks can be effective in increasing users’ adherence and engagement with the wearable trackers [37]. However, this study had a couple of limitations—it included a small number of users, as well as nonusers of wearable trackers; and it examined Web-based social networks as a stand-alone feature, not integrated with the trackers. In contrast, our study provided participants with an integrated intervention, including mHealth and social networking components, which allowed us to explore the informed perspectives of participants who used these technologies for 6 months.

### Individual-Level Behavior Change Techniques

Our users indicated that goal setting, self-monitoring of behavior, and feedback on behavior could encourage them to engage in physical activity, which is in line with previous qualitative studies [18,19]. Indeed, these 3 self-regulatory techniques have demonstrated the effectiveness in physical activity interventions [11] and may work in synergy—to maximize the effects of goal setting, people may need to self-monitor and receive feedback, which allows them to see their progress in relation to their goals and change their strategies if necessary [38].

In addition, previous research has suggested the need to examine which type of goal is best for motivating individuals to be more active and how technologies can best support monitoring those goals and providing feedback. The literature seems to suggest that small goals (described as “graded tasks” in the Coventry, Aberdeen, and London—Refined taxonomy [10]) are more effective for long-term engagement compared with larger and harder to achieve goals [39]. For example, Fitbit provides users with small goals of taking 250 steps per hour, which then facilitates the achievement of the daily goal of 10,000 steps [23]. It is worth noting the importance of real-time self-monitoring and consistent feedback for the success of this “small goals” approach [23], underlining implications for the design of mobile apps and wearable trackers.

### Social Networks and Social Features

This study emphasized the role of social comparison, familiarity, and similarity with other users in a social networking intervention. First, our participants revealed different preferences regarding social comparison. This finding is in line with previous research, where it has been demonstrated that individual preferences might depend on their tendency to make upward or downward comparisons [40]. Specifically, previous studies have illustrated that some people seek social comparison to self-improve [33], and thus, upward comparison may reinforce positive fitness behavior by making it seem normative or even rewarding [41,42]. For others, instead of seeking feedback about themselves, they want to create and maintain a positive self-image, and thus, prefer to make a downward comparison [33,42]. Taken as a whole, this finding suggests that a one-size-fits-all approach to social comparison is unlikely to suit all users, and thus, social comparison needs to be tailored to each individual.

Second, *familiarity* and *similarity* were found to be important factors in a social networking intervention for physical activity. The importance of familiarity seems to be in line with previous literature, where researchers have demonstrated that existing social networks can greatly influence individual health behaviors [43,44], leveraging social support and potentially increasing the intervention effectiveness [17,40,45-47]. Research has shown that strategies involving new networks might not be as effective as ones capitalizing on existing connections [46,47], which suggests that fitness technology may be most effective when groups of people who know one another have access to the same device or app [23]. Thus, allowing study participants to invite friends and family to join an app may increase the real-world effectiveness of these interventions [40], despite potential problems of contamination.

Furthermore, this study showed that similarity is important for motivation and engagement, highlighting the role of homophily (ie, the tendency of people to bond with alike individuals) [34]. Notably, previous research has indicated that social networks structured on the basis of homophily lead to higher adoption of healthy behaviors [48]. Moreover, it has been suggested that when people with similar interests interact to achieve a shared goal, they can provide each other with support and companionship in the activity, and thus, reduce the perceived costs of adopting a new exercise routine [46,49]. Taken together, these findings highlight the benefits of leveraging homophily to foster collective efficacy and improve physical activity.

### Technology As a Platform to Bring Together Individual and Social Levels

Through automation and personalization, multiple modes and features of technology can work synergistically to deliver a physical activity intervention with both individual and social factors [37,50,51]. Thus, the integration of multiple mHealth technologies can automate several aspects of health management, reducing the burden on users. Furthermore, many users suggested the importance of personalized features within the intervention. Indeed, a one-size-fits-all approach is unlikely to satisfy many needs and wants of users [52], which emphasizes the need to consider individual lifestyles and preferences when designing interventions.

### Strengths and Limitations

This study has several strengths. We interviewed users after 6 months of experiencing the intervention, ensuring that our sample had an informed perspective. The combination of individual interviews and focus groups enabled us to capture both individual perspectives and social dynamics in a group setting, which are essential aspects to understand in a social networking intervention. The findings of this paper must be interpreted in light of some limitations. First, study recruitment was limited to a university setting with a young age group. Though the main purpose of qualitative studies is not to make generalizable claims [53], future research with a diverse sample could explore other contextual factors related to behavioral informatics interventions (eg, an older age group might encounter different barriers and facilitators of a mobile social networking app). Second, as this was part of a feasibility study, the technology used was at a prototype stage and not yet extensively tested. Finally, despite our engagement efforts, we were not able to interview participants who dropped out of the study—they might have different perspectives on the facilitators and barriers of the intervention.

### Implications for Future Research

This study highlights several important implications, including suggestions on the intervention design and new research avenues. Interventions for physical activity promotion should consider offering goal setting, self-monitoring, and feedback as a bundle, as these techniques have been shown to be both effective and acceptable to end users. Consequently, the design of mobile apps and wearable trackers need to effectively assist with real-time self-monitoring and provide consistent feedback to enable the achievement of goals [23]. In addition, the potential of social behavior change techniques (eg, social comparison) should be further explored, and aspects of leveraging existing social ties and homophily could be considered in constructing a social network intervention for physical activity. Questions remain about the cost-effectiveness of wearable trackers and mobile apps as a public health initiative, opening up new possibilities for future health economics research and public health programs [23,54].

Furthermore, this study highlights the importance of *personalization*. By identifying users’ behavioral patterns and preferences, researchers can design and deliver interventions at the right time, using the right channel and tone, and the most relevant content or services [55,56]. Future studies should use innovative study designs to determine which intervention components are effective, what is the optimal sequence for delivering these components, and which tailoring variables should be used [23,57].

### Conclusions

This study provides insights into the individual, social, and technological factors that influence user engagement with a mobile social networking app for physical activity promotion. Our findings reveal that self-regulatory behavior change techniques seem to be a necessary element in these interventions, and that aspects related to social comparison, existing social ties, and homophily should be considered in the development of the social network component. Future research should adopt innovative research designs to evaluate the effectiveness of these different components, as well as investigate the delivery of personalized interventions.

## Appendix

Multimedia Appendix 1 COnsolidated criteria for REporting Qualitative research checklist.

Multimedia Appendix 2 Screenshots of the fit.healthy.me mobile app.

Multimedia Appendix 3 Interview guides.

## Abbreviations

BMI: body mass index
mHealth: mobile health
