# Supplementary material for: Using a Mobile Social Networking App to Promote Physical Activity: A Qualitative Study of Users’ Perspectives
Source: J Med Internet Res. 2018 Dec 21;20(12):e11439. doi: 10.2196/11439 (PMC6320410; doi:10.2196/11439)
Supplement: Multimedia Appendix 1 [file jmir_v20i12e11439_app1.pdf]

Appendix 1: Consolidated criteria for reporting qualitative studies (COREQ): 32-item checklist

Developed from:

Tong A, Sainsbury P, Craig J. Consolidated criteria for reporting qualitative research (COREQ): a 32-item checklist for interviews and focus groups. *International Journal for Quality in Health Care*. 2007. Volume 19, Number 6: pp. 349 – 357

**No. Item**	**Guide questions/description**	**Reported on Section**
**Domain 1: Research team and reflexivity**		
Personal Characteristics		
1. Interviewer/facilitator	Which author/s conducted the interview or focus group?	Authors' Contributions
2. Credentials	What were the researcher's credentials? E.g. PhD, MD	Authors' names and titles
3. Occupation	What was their occupation at the time of the study?	Authors' affiliation
4. Gender	Was the researcher male or female?	Authors' names and titles
5. Experience and training	What experience or training did the researcher have?	Authors' names and titles, Methods/Interview Procedure section
Relationship with participants		
6. Relationship established	Was a relationship established prior to study commencement?	No
7. Participant knowledge of the interviewer	What did the participants know about the researcher? e.g. personal goals, reasons for doing the research	Reasons for doing research - Methods/Interview Procedure section
8. Interviewer characteristics	What characteristics were reported about the interviewer/facilitator? e.g. Bias, assumptions, reasons and interests in the research topic	All the authors had a positive attitude towards mobile health technologies and online social networks, but the authors strived to remain neutral in the conversations with participants.

**Domain 2: study design**		
Theoretical framework		
9. Methodological orientation and Theory	What methodological orientation was stated to underpin the study? e.g. grounded theory, discourse analysis, ethnography, phenomenology, content analysis	Thematic analysis – Methods/Data Management and Analysis
Participant selection		
10. Sampling	How were participants selected? e.g. purposive, convenience, consecutive, snowball	Purposive – Methods/Study Setting and Participants
11. Method of approach	How were participants approached? e.g. face-to-face, telephone, mail, email	Methods/Study Setting and Participants
12. Sample size	How many participants were in the study?	Methods/Study Setting and Participants
13. Non-participation	How many people refused to participate or dropped out? Reasons?	Methods/Study Setting and Participants
Setting		
14. Setting of data collection	Where was the data collected? e.g. home, clinic, workplace	Methods/Interview Procedure section
15. Presence of non-participants	Was anyone else present besides the participants and researchers?	No
16. Description of sample	What are the important characteristics of the sample? e.g. demographic data, date	Table 2
Data collection		
17. Interview guide	Were questions, prompts, guides provided by the authors? Was it pilot tested?	Methods/Interview Procedure section
18. Repeat interviews	Were repeat interviews carried out? If yes, how many?	Methods/Interview Procedure section
19. Audio/visual recording	Did the research use audio or visual recording to collect the data?	Audio
20. Field notes	Were field notes made during and/or after the inter view or focus group?	Methods/Interview Procedure section

21. Duration	What was the duration of the interviews or focus group?	Methods/Interview Procedure section
22. Data saturation	Was data saturation discussed?	Methods/Interview Procedure section
23. Transcripts returned	Were transcripts returned to participants for comment and/or correction?	No
**Domain 3: analysis and findings**		
Data analysis		
24. Number of data coders	How many data coders coded the data?	Methods/Data Management and Analysis
25. Description of the coding tree	Did authors provide a description of the coding tree?	N/A
26. Derivation of themes	Were themes identified in advance or derived from the data?	Methods/Data Management and Analysis
27. Software	What software, if applicable, was used to manage the data?	Methods/Data Management and Analysis
28. Participant checking	Did participants provide feedback on the findings?	No
Reporting		
29. Quotations presented	Were participant quotations presented to illustrate the themes/findings? Was each quotation identified? e.g. participant number	Textboxes 1, 2, 3
30. Data and findings consistent	Was there consistency between the data presented and the findings?	Yes, there was. Results & Discussion
31. Clarity of major themes	Were major themes clearly presented in the findings?	Yes, they were. Results & Discussion
32. Clarity of minor themes	Is there a description of diverse cases or discussion of minor themes?	Discussion of major and minor themes Results & Discussion
