# Supplementary material for: Using a Mobile Social Networking App to Promote Physical Activity: A Qualitative Study of Users’ Perspectives
Source: J Med Internet Res. 2018 Dec 21;20(12):e11439. doi: 10.2196/11439 (PMC6320410; doi:10.2196/11439)
Supplement: Multimedia Appendix 2 [file jmir_v20i12e11439_app2.pdf]

Appendix 2: Screenshots of the fit.healthy.me app

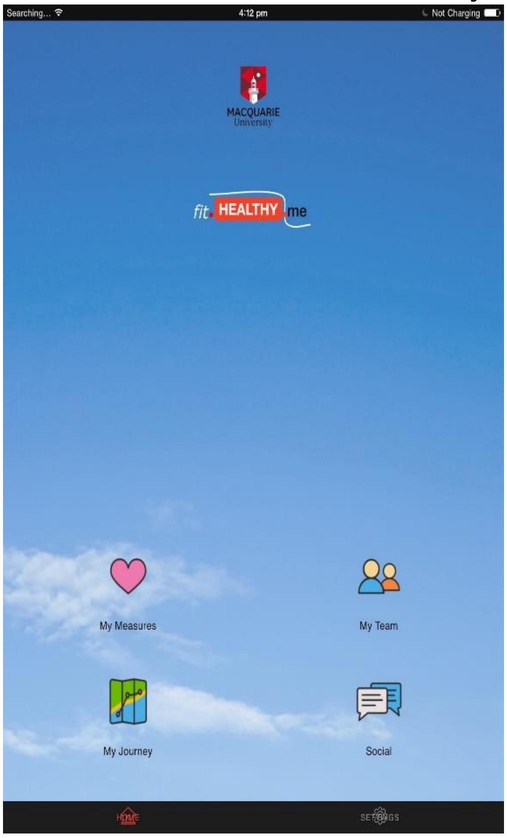

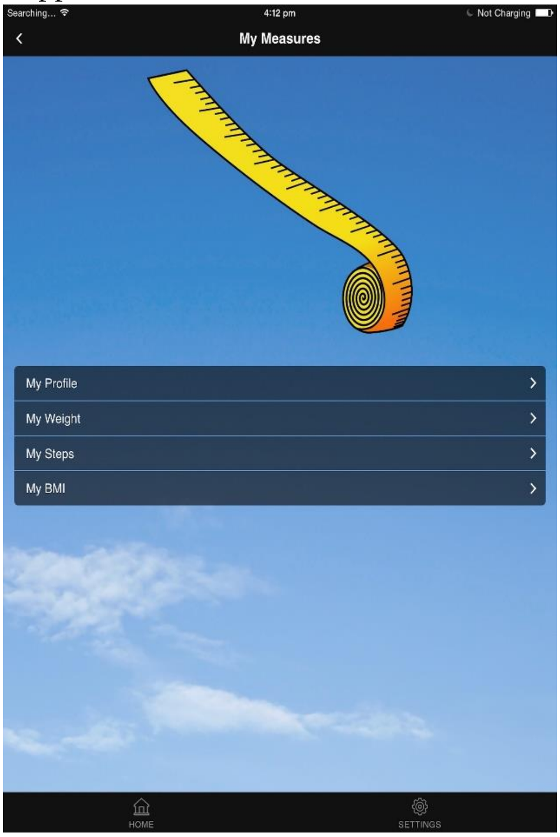

a) Homepage

b) My measures

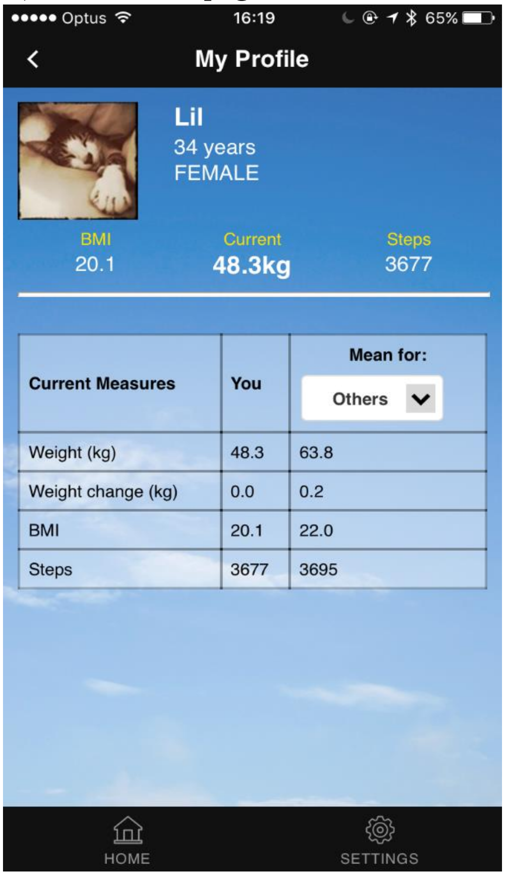

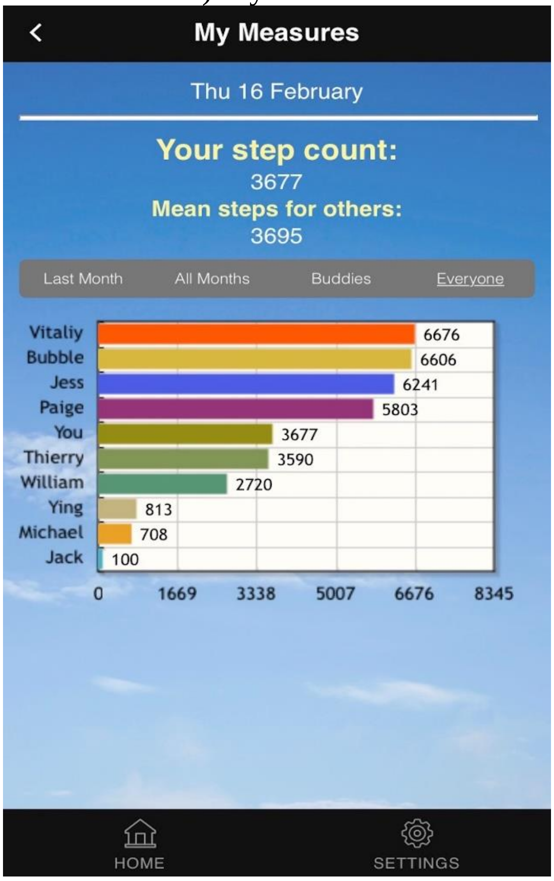

c) Social comparison features
