# Supplementary material for: Using a Mobile Social Networking App to Promote Physical Activity: A Qualitative Study of Users’ Perspectives
Source: J Med Internet Res. 2018 Dec 21;20(12):e11439. doi: 10.2196/11439 (PMC6320410; doi:10.2196/11439)
Supplement: Multimedia Appendix 3 [file jmir_v20i12e11439_app3.pdf]

**Appendix 3. Interview guides at pre-intervention sessions and post-intervention sessions**

**Pre-intervention sessions**

1. **Physical activity**
   a. What helps you to exercise regularly?
   b. What prevents you from exercising regularly?
2. **Weight management**
   a. What helps you to maintain a healthy weight?
   b. What prevents you from maintaining a healthy weight?
3. **Wearable devices**
   a. What do you think are possible advantages of monitoring physical activity and weight? What are the disadvantages?
   b. Are you using, or have you used in the past, any wearable/tracking devices to monitor your physical activity and weight? If yes, which ones?
   c. What do you think are possible advantages of using wearable/tracking devices to monitor your health? And disadvantages?
4. **Mobile apps**
   a. Do you use, or have you used in the past, any mobile apps to monitor your health or to track lifestyle/fitness activities? If yes, which ones?
   b. What do you think are possible advantages of using a mobile app to monitor your health? And disadvantages?
5. **Social features**
   a. Do you use any social networking sites? Have you ever used social networking sites for health purposes? (e.g. search health information, participate in fitness or health-related groups)
   b. What do you think are possible advantages of using social network features to facilitate weight management and physical activity? And disadvantages?
6. Is there any comment you want to make?

**Post-intervention sessions**

1. How did you find the experience of participating in the study?
2. **Wearable devices**
    a. What were the benefits of using the wearable device to monitor your activity and weight?
    b. What were the disadvantages of using the wearable device to monitor your physical activity and weight? Prompts: ease-of-use; convenience; integration in daily routine
    c. In your previous interview, you mentioned that … Has your opinion about the wearable device changed after using it?
    d. What device (Fitbit tracker/scale) do you choose to keep? Why?

3. **Health apps**

a. What were the benefits of using the fit.healthy.me app to monitor your physical activity and weight?
b. What were the disadvantages of using the fit.Healthy.me app?
c. In your previous interview, you mentioned that … Has your opinion about using the app changed after using it?

4. **Social features**
    a. What were the benefits of the social components in this intervention? (Prompts: tables, graphs to compare yourself, the social forum)
    b. What were the disadvantages of the social components?
    c. In your previous interview, you mentioned that … Has your opinion about using social media to help physical activity and weight management changed after using it?
    d. Did you have any social connections with other people who are in the study? (Did you know them before or after the study?)
5. **Suggestion:**
    a. **Keep using**: We noticed that you were really engaged with the study (using the app, scale and tracker). What helped you to be so engaged?

OR

b. **Stop using**: We noticed that you stopped using the app/tracker after [X months]. Why did you stop? What could we have done to help you stay engaged?

c. Do you have **any suggestions** about additional aspects of the app or the devices that could be helpful in terms of monitoring activity and weight?
